# First-line treatments in EGFR-mutated advanced non-small cell lung cancer: A network meta-analysis

**DOI:** 10.1371/journal.pone.0223530

**Published:** 2019-10-03

**Authors:** Hongwei Zhang, Jun Chen, Tingting Liu, Jun Dang, Guang Li

**Affiliations:** 1 Department of Radiation Oncology, The First Hospital of China Medical University, Shenyang, China; 2 Department of Radiation Oncology, Shenyang Chest Hospital, Shenyang, China; 3 Department of Radiation Oncology, Anshan Cancer Hospital, Anshan, China; George Washington University Milken Institute of Public Health, UNITED STATES

## Abstract

**Background:**

It remains unknown which is the optimal first-line treatment regimen for patients with advanced epidermal growth factor receptor (EGFR)-mutated non-small cell lung cancer (NSCLC). We performed a network meta-analysis to address this important issue.

**Methods:**

PubMed, Embase, Cochrane Library, Web of Science and major international scientific meetings were searched for relevant randomized controlled trials (RCTs). Progression-free survival (PFS) data was the primary outcome of interest, and overall survival (OS) and serious adverse events (SAEs) were the secondary outcomes of interests, reported as hazard ratio (HR) or odds ratio (OR) and 95% confidence intervals (CIs).

**Results:**

25 RCTs with a total of 5005 patients randomized to receive seven treatments were included in the meta-analysis. Third-generation tyrosine kinase inhibitor (TKI) (osimertinib) and first-generation TKIs (F-TKIs) in combination with chemotherapy (F-TKIs+CT) were more effective than F-TKIs alone in terms of PFS (HR = 0.46, 95% CI: 0.22–0.93; *P* = 0.031 and HR = 0.62, 95% CI: 0.39–0.98; *P* = 0.041) and OS (HR = 0.63, 95% CI: 0.43–0.91; *P* = 0.014 and HR = 0.73, 95% CI: 0.57–0.92; *P* = 0.008). Second-generation TKIs (S-TKIs) showed significant OS advantage over F-TKIs (HR = 0.83, 95% CI: 0.70–0.99; *P* = 0.04). Based on treatment ranking in terms of PFS and OS, osimertinib had the highest probability of being the most effective treatment (89% and 86%) and with the best tolerability. F-TKIs+CT was ranked the second-most effective regimen, but with relatively high risk of SAEs.

**Conclusions:**

Osimertinib seemed to be the most preferable first-line treatment in advanced EGFR-mutated NSCLC. However, limitations of the study including a single RCT investigating osimertinib and immature OS data need to be considered.

## Introduction

Non-small cell lung cancer (NSCLC) remains the leading cause of cancer-associated mortality globally [1–2], and approximately 15% to 50% of NSCLC patients have an activating epidermal growth factor receptor (EGFR) mutation [3]. First-generation tyrosine kinase inhibitors (F-TKIs) (gefitinib, erlotinib, or icotinib) have consistently shown a progression-free survival (PFS) benefit compared to chemotherapy (CT) in first-line of treatment of advanced EGFR-mutated NSCLC [4–10]. Recently, the survival differences between F-TKIs, second-generation TKIs (S-TKIs) (afatinib or dacomitinib), and third-generation TKI (osimertinib) have been investigated in a number of trials [11–15]. Most of the trials have demonstrated the benefit of S-TKIs and osimertinib over F-TKIs in previously treated advanced EGFR-mutated NSCLC. Promising results also have been reported for F-TKIs in combination with CT (F-TKIs+CT) [16–19] or Bevacizumab (F-TKIs+Bev) [20–22]. However, direct comparison trials between S-TKIs, T-TKIs, and combination regimens involving TKIs are still lacking, and therefore, there are still unresolved questions around which is the optimal first-line treatment for patients with advanced EGFR-mutated NSCLC.

Two previous network meta-analyses [23–24] have evaluated first-line treatments in advanced EGFR-mutated NSCLC. However, first-line treatment of osimertinib has not been assessed in Lin et al’ study [23], while first-line treatment of combination regimens involving TKIs has not been evaluated in Batson et al’ study [24]. Moreover, since the two meta-analyses, several available RCTs [19, 22, 25] have been newly published. Thus, we performed a novel network meta-analysis, attempting to identify the most preferable first-line treatment regimen in patients with advanced EGFR-mutated NSCLC.

## Methods

This study adhered to the Preferred Reporting Items for Systematic Reviews and Meta-Analyses (PRISMA) criteria [26] (S1 Table).

### Literature search strategy

Two investigators independently searched PubMed, Embase, Cochrane Library, Web of Science, and major international scientific meetings (American Society of Clinical Oncology, European Society for Medical Oncology, and World Conference on Lung Cancer) for the available trials published before March 1, 2019. The detailed strategies are shown in S2 Table.

### Inclusion and exclusion criteria

Studies meeting the following criteria were included: (1) types of studies: randomized controlled trials (RCTs); (2) types of participants: advanced EGFR-mutated NSCLC patients; (3) types of interventions: at least one intervention was a TKI (F-TKIs, S-TKIs, or osimertinib), alone or in combination with other types of treatments; and (4) outcome: reported PFS or overall survival (OS) data. Studies which failed to meet the above criteria were excluded from the network meta-analysis.

### Data extraction

Two investigators extracted the following data from each study independently: first author or RCT name, year of publication, duration of RCT, region, interventions, numbers of patients, hazard ratios (HRs) and their 95% confidence intervals (CIs) of PFS and OS, and odds ratios (ORs) and their 95% CIs of serious adverse events (SAEs). Crude HRs with 95% CIs for PFS and OS were either extracted directly from the original reports or calculated by the Kaplan–Meier curves based on the methods of Parmar et al. [27] and Tierney et al. [28]. Data on the overall numbers of patients with SAEs were directly extracted if they were reported in the published article. If only the numbers of individual SAEs were reported separately in articles, we pooled all numbers of them to represent the overall numbers of SAEs.

### Quality assessment

Cochrane risk of bias tool [29] was used to evaluate the methodological quality of RCTs, which includes the following five domains: sequence generation, allocation concealment, blinding, incomplete data, and selective reporting. A RCT was rated as “low risk of bias” if all key domains indicated as low risk, was rated as “high risk of bias” if one or more key domains indicated as high risk, and was judged to be “unclear risk of bias” when more than three domains indicated as unclear risk.

### Statistical analysis

The primary outcome was PFS, and the secondary outcomes were OS and SAEs. For direct comparisons, standard pairwise meta-analysis (PWMA) was performed. The heterogeneity between studies was assessed by chi-square (χ^2^) and *I*-square (*I*^2^) tests. A *P* value <0.10 or *I*^2^ > 50% was considered significant heterogeneity existing, and a random-effects analysis model was used; otherwise, a fixed-effects model was used. PWMA was performed using the software Review Manager 5.3 (Cochrane Collaboration, Oxford, UK).

The Bayesian network-meta analysis (NMA) for all outcomes were performed in a random-effect model [Generalized Linear Model (GLM)] using Markov chain Monte Carlo methods [30–31] in JAGS and the GeMTC package in R (https://drugis.org/software/r-packages/gemtc). OS and PFS were analyzed with GLM with a normal likelihood incorporating log hazard ratio statistics from individual trials to calculate HR between competing treatments. Count statistics of SAEs was analyzed with GLM with a binomial likelihood to calculate relative treatment effects expressed as OR between different treatments. For each outcome measure, four independent Markov chains were simultaneously run for 20,000 burn-ins and 100,000 inference iterations per chain to obtain the posterior distribution. The traces plot and Brooks-Gelman-Rubin method were used to assess the convergence of model [32]. Treatment effects were estimated by HR/OR and corresponding 95% CI. Network consistency was assessed with node-split models by statistically testing between direct and indirect estimates within treatment loop [33]. To rank probabilities of all available treatments, the surfaces under the cumulative ranking curve (SUCRAs) were calculated [34]. SUCRA equals one if the treatment is certain to be the best and zero if it’s certain to be the worst [34]. To jointly compare the efficacy and tolerability of each treatment and to assess their benefit-risk ratios, we ranked them based simultaneously on the SUCRA value of PFS and tolerability (1-SUCRA_SAEs_) in the ranking plot. Lastly, comparison-adjusted funnel plot was used to detect the presence of small-study effects or publication bias.

## Results

### Literature search results and characteristics of included studies

**Fig 1:**
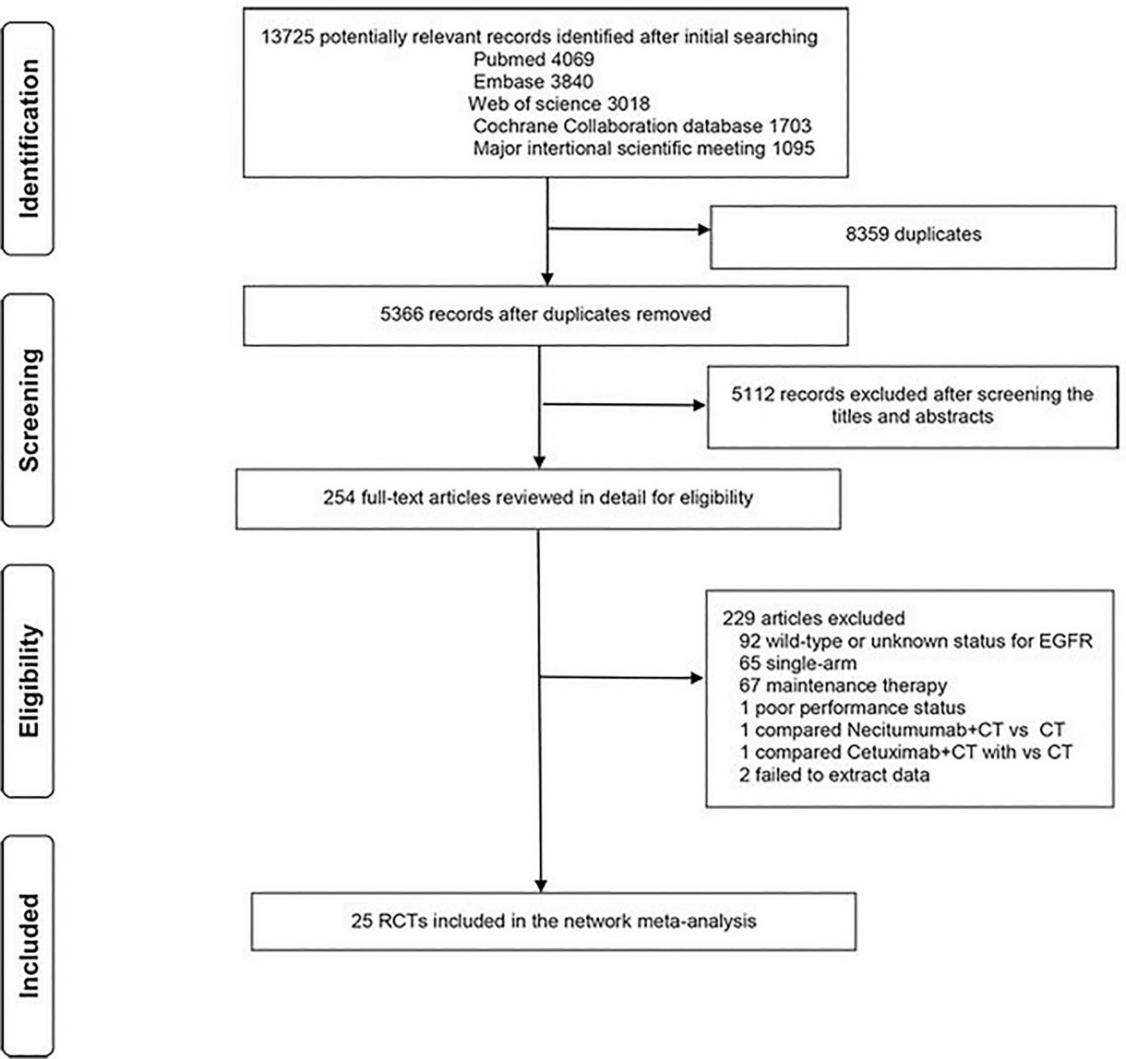
Literature search and selection.

Fig 1 shows the flow diagram for study selection. The preliminary literature search identified 13725 studies. 13471 of them were excluded after removing the duplicates and an abstract review. The remaining 254 studies were screened through a full-text review for further eligibility. All relevant references were also reviewed. Finally, 25 RCTs [4–22, 25, 35–47] with 5005 patients were included in the network meta-analysis and compared seven treatments including F-TKIs, S-TKIs, osimertinib, CT, F-TKIs+CT, F-TKIs+Bev, and F-TKIs+Linsitinib (F-TKIs+Lin). The clinical characteristics are shown in Table 1.

**Table 1:** Clinical characteristics of included trials.

Trial	Design	Time	Region	Primary	Treatment	Sample	Median follow-up
		Range		Endpoint		Size	(months)
NEJ002/2010[4–5]	III	2006–2009	Multicenter	PFS	F-TKIs	114	>17
					CT	114	
WJTOG3405/2010[6–7]	III	2006–2009	Japan	PFS	F-TKIs	86	34
					CT	86	
EURTAC/2012[8]	III	2007–2011	Multicenter	PFS	F-TKIs	86	18.9
					CT	87	14.4
OPTIMAL/2011[9–10]	III	2011–2014	China	PFS	F-TKIs	82	25.9
					CT	72	
LUX-Lung7/2016[11–12]	II	2011–2013	Multicenter	PFS	S-TKIs	160	42.6
					F-TKIs	159	
ARCHER1050/2017[13–14]	III	2013–2015	Asian	PFS	S-TKIs	227	22.1
					F-TKIs	225	
FLAURA/2017[15]	III	2014–2016	Multicenter	PFS	Osimertinib	279	15
					F-TKIs	277	9.7
FASTACT-2/2013[16]	II	2009–2010	Asian	PFS	F-TKIs+CT	49	27.6
					CT	48	
Yu/2014[17]	II	2010–2012	China	ORR	F-TKIs+CT	14	NR
					CT	18	
Cheng/2016[18]	II	2012–2013	Asian	PFS	F-TKIs+CT	126	NR
					F-TKIs	65	
NEJ009/2018[19]	III	2011–2014	Japan	PFS	F-TKIs+CT	172	NR
					F-TKIs	170	
JO25567/2014[20–21]	II	2011–2012	Japan	PFS	F-TKIs+Bev	75	25.9
					F-TKIs	77	27
NEJ026/2018[22]	III	NR	Japan	PFS	F-TKIs+Bev	112	12.4
					F-TKIs	112	
CONVINCE/2017[25]	III	2013–2014	China	PFS	F-TKIs	148	18
					CT	137	15.7
IPASS/2009[35–36]	III	2005–2008	Asian	PFS	F-TKIs	132	17
					CT	129	
TORCH/2012[37]	III	2006–2009	Italy, Canada	OS	F-TKIs	19	24.3
					CT	20	
Chen/2012[38]	II	2007–2008	China	PFS	F-TKIs	9	NR
					CT	15	
ENSURE/2015[39]	III	2011–2012	Asian	PFS	F-TKIs	110	28.9
					CT	107	27.1
Han/2012[40]	III	2005–2007	Korea	OS	F-TKIs	26	35
					CT	16	
LUX-Lung3/2013[41–42]	III	2009–2011	Multicenter	PFS	S-TKIs	230	41
					CT	115	
LUX-Lung6/2014[42–43]	III	2010–2011	Asian	PFS	S-TKIs	242	33
					CT	122	
Hirsch/2011[44]	II	2007–2008	Multicenter	PFS	F-TKIs+CT	6	NR
					F-TKIs	9	
CALGB30406/2012[45]	II	2005–2009	United States	PFS	F-TKIs+CT	33	38
					F-TKIs	33	
TRIBUTE/2005[46]	III	2001–2002	United States	OS	F-TKIs+CT	93	NR
					CT	74	NR
Leighl/2017[47]	II	NR	Multicenter	PFS	F-TKIs+Lin	44	NR
					F-TKIs	44	

Abbreviations: PFS, progression-free survival; OS, overall survival; ORR, overall response rate; NR, not reported; TKIs, tyrosine kinase inhibitor; F, first-generation; S, second-generation; Bev, bevacizumab; CT, chemotherapy; Lin, Linsitinib.

### Assessment of included trial

The demographic characteristics of involved patients were generally well-balanced between different trials and different arms within each trial (see S3 Table). Median age ranged from 56 to 68 years. 22–44% of patients were male; and 0–14% were with squamous cell carcinoma. Most of patients were with Stage IV disease (73.2–100%), except patients included in WJTOG3405 trial (47.7%) [6–7]. EGFR mutations were mainly exon 19 deletions and 21 deletions mutations. In eleven studies, details of baseline demographic characteristics were not stated [16–17, 35–38, 40, 44–46]. The risk of bias in included RCTs was summarized in S1 Fig. Four RCTs [4–5, 17, 19, 22] were judged to be unclear risk of bias, as they had more than three domains indicating as unclear risk. The remaining RCTs were judged to be low risk of bias. No trial was rated with a high risk of bias. Funnel plot analysis in term of PFS did not indicate any evident risk of publication bias (S2 Fig).

### Conventional pairwise meta-analysis

Results of individual trials are shown in S4 table. Results of PWMA are shown in Table 2. In terms of PFS, S-TKIs (HR = 0.65, 95% CI: 0.54–0.77, *P*_heterogeneity_ = 0.23), F-TKIs+CT (HR = 0.57, 95% CI: 0.47–0.70, *P*_heterogeneity_ = 0.22), and F-TKIs+Bev (HR = 0.56, 95% CI: 0.43–0.74, *P*_heterogeneity_ = 0.55) were more effective than F-TKIs. With regard to OS, S-TKIs showed significant advantage over F-TKIs (HR = 0.81, 95% CI: 0.67–0.97, *P*_heterogeneity_ = 0.52). As for overall SAEs, S-TKIs (OR = 2.29, 95% CI: 1.69–3.12, *P*_heterogeneity_ = 0.58), F-TKIs+CT (OR = 3.79, 95% CI: 2.58–5.56, *P*_heterogeneity_ = 0.58), and F-TKIs+Bev (OR = 4.05, 95% CI: 1.04–15.86, *P*_heterogeneity_ = 0.009) were more likely to cause SAEs than F-TKIs.

**Table 2:** Results of direct comparisons.

Outcome	Treatment	No. of	No. of	HR/OR(95%CI)	Heterogeneity	
		studies	patients		I^2^	P
PFS	S-TKIs vs F-TKIs	2	771	HR 0.65(0.54–0.77)	31%	0.23
	F-TKIs+CT vs F-TKIs	4	614	HR 0.57(0.47–0.70)	32%	0.22
	F-TKIs+Bev vs F-TKIs	2	376	HR 0.56(0.43–0.74)	0	0.55
	F-TKIs vs CT	10	1595	HR 0.46(0.29–0.72)	93%	<0.001
	F-TKIs+CT vs CT	2	129	HR 0.24(0.16–0.37)	0	0.75
	S-TKIs vs CT	2	709	HR 0.40(0.20–0.83)	90%	0.001
OS	S-TKIs vs F-TKIs	2	771	HR 0.81(0.67–0.97)	0	0.52
	F-TKIs vs CT	9	1423	HR 1.01(0.88–1.15)	0	0.87
	S-TKIs vs CT	2	709	HR 0.91(0.74–1.10)	0	0.78
	F-TKIs+CT vs F-TKIs	2	408	HR 0.70(0.54–0.92)	0	0.93
	F-TKIs+CT vs CT	2	264	HR 0.71(0.35–1.46)	78%	0.03
SAEs	S-TKIs vs F-TKIs	2	771	OR 2.29(1.69–3.12)	0	0.58
	F-TKIs+CT vs F-TKIs	2	533	OR 3.79(2.58–5.56)	0	0.58
	F-TKIs+Bev vs F-TKIs	2	376	OR 4.05(1.04–15.86)	85%	0.009
	F-TKIs vs CT	6	1229	OR 0.30(0.21–0.43)	48%	0.09
	S-TKIs vs CT	2	709	OR 0.68(0.21–2.22)	92%	<0.001

Abbreviations: No., number; HR, hazard ratio; CI, confidence interval; OR, odds ratio; PFS, progression-free survival; OS, overall survival; SAEs, serious adverse events; TKIs, tyrosine kinase inhibitor; F, first-generation; S, second-generation; Bev, bevacizumab; CT, chemotherapy.

### Network meta-analysis

**Fig 2:**
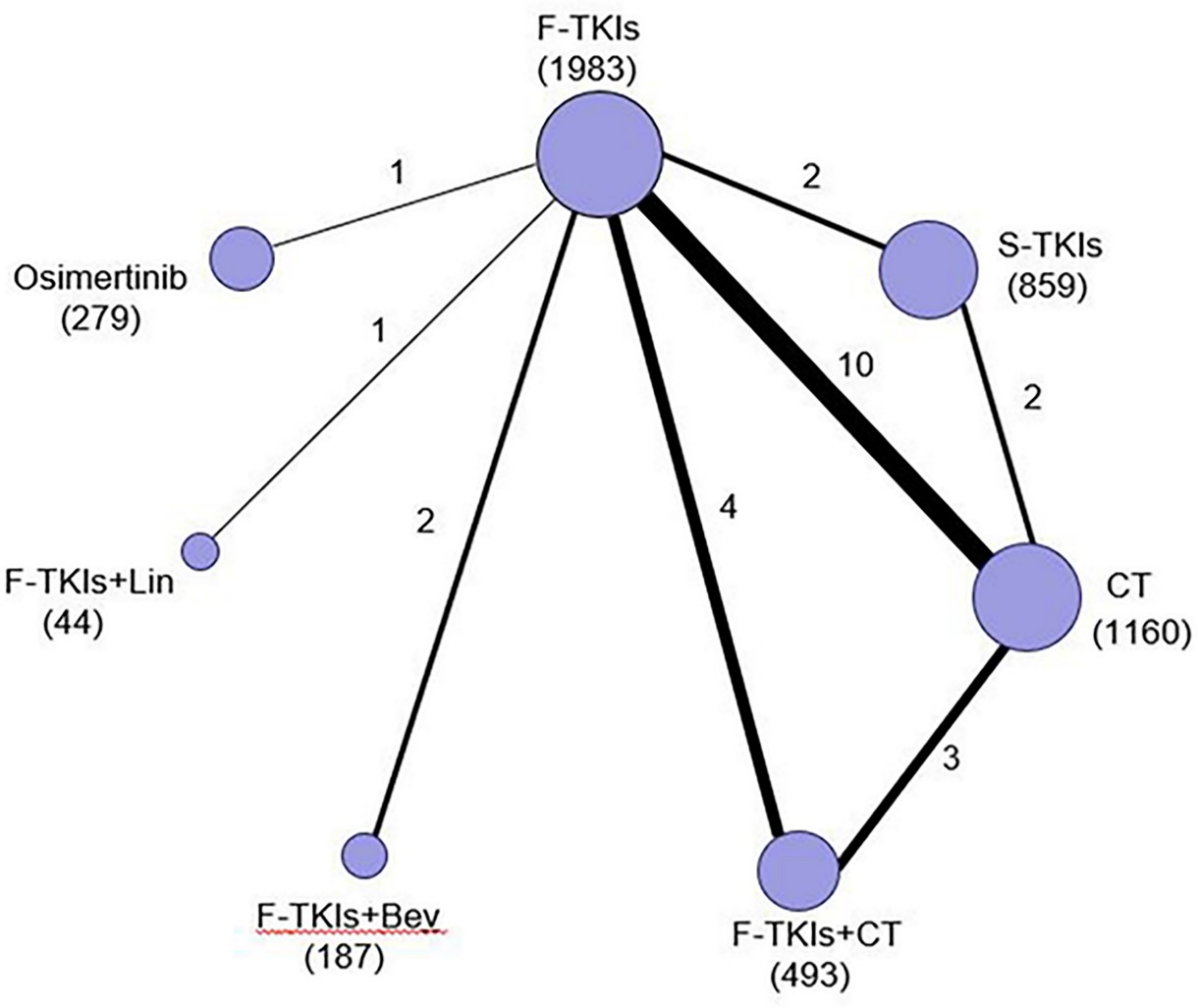
Network of eligible comparisons. The size of the nodes is proportional to the number of patients (in parentheses) randomized to receive the treatment. The width of the lines is proportional to the number of trials (beside the line) comparing the connected treatments. TKIs, tyrosine kinase inhibitor; F, first-generation; S, second-generation; Bev, bevacizumab; CT, chemotherapy; Lin, Linsitinib.

The network plot established for NMA is shown in Fig 2. Results of the NMA were presented in Table 3. Osimertinib and F-TKIs+CT were more effective than F-TKIs in terms of PFS (HR = 0.46, 95% CI: 0.23–0.93; *P* = 0.031 and HR = 0.62, 95% CI: 0.39–0.98; *P* = 0.041) and OS (HR = 0.63, 95% CI: 0.43–0.91; *P* = 0.014 and HR = 0.73, 95% CI: 0.57–0.92; *P* = 0.008). S-TKIs showed significant OS advantage over F-TKIs (HR = 0.83, 95% CI: 0.70–0.99; *P* = 0.04). Other comparisons among TKIs based regimens in terms of PFS or OS did not produce statistically significant differences. With regard to overall SAEs, osimertinib showed significantly lower risk of causing SAEs in comparison to each TKIs based regimens except F-TKIs; F-TKIs had significantly lower risk of causing SAEs than each TKIs based regimens except osimertinib. No significant differences were observed in other comparisons between TKIs based regimens in terms of SAEs.

**Table 3:** Results of network meta-analysis.

a. Hazard ratios(HR) with 95% confidence interval (CI) for progression-free survival (PFS)						
Osimertinib						
0.75(0.28–2.0)	F-TKIs+CT					
0.74(0.25–2.3)	0.99(0.45–2.2)	F-TKIs+Bev				
0.60(0.22–1.6)	0.80(0.43–1.5)	0.80(0.36–1.8)	S-TKIs			
**0.46(0.23–0.93)**	**0.62(0.39–0.98)**	0.62(0.32–1.2)	0.77(0.48–1.2)	F-TKIs		
0.34(0.09–1.3)	0.45(0.15–1.4)	0.46(0.13–1.5)	0.57(0.18–1.7)	0.73(0.26–2.0)	F-TKIs+Lin	
**0.20(0.10–0.43)**	**0.27(0.18–0.41)**	**0.27(0.15–0.50)**	**0.34(0.23–0.50)**	**0.44(0.35–0.56)**	0.61(0.24–1.52)	CT
b. Hazard ratios(HR) with 95% confidence interval (CI) for overall survival(OS)						
Osimertinib						
0.87(0.56–1.3)	F-TKIs+CT					
0.78(0.43–1.4)	0.90(0.54–1.5)	F-TKIs+Bev				
0.76(0.50–1.1)	0.87(0.66–1.2)	0.97(0.60–1.6)	S-TKIs			
**0.63(0.43–0.91)**	**0.73(0.57–0.92)**	0.81(0.51–1.3)	**0.83(0.70–0.99)**	F-TKIs		
0.82(0.25–2.8)	0.95(0.29–3.1)	1.1(0.31–3.7)	1.1(0.34–3.5)	1.3(0.42–4.2)	F-TKIs+Lin	
**0.65(0.46–0.93)**	**0.76(0.61–0.95)**	0.84(0.54–1.31)	0.87(0.75–1.01)	1.04(0.93–1.17)	0.80(0.25–2.58)	CT
c. Odds ratios (OR) with 95% confidence interval (CI) for serious adverse events (SAEs)						
Osimertinib						
**0.18(0.06–0.56)**	F-TKIs+CT					
**0.18(0.06–0.58)**	1.01(0.36–2.82)	F-TKIs+Bev				
**0.30(0.11–0.81)**	1.62(0.69–3.84)	1.61(0.65–3.97)	S-TKIs			
0.67(0.28–1.61)	**3.68(1.83–7.41)**	**3.65(1.72–7.74)**	**2.27(1.38–3.73)**	F-TKIs		
**0.19(0.04–0.85)**	1.03(0.25–4.23)	1.03(0.24–4.31)	0.64(0.17–2.39)	**0.28(0.08–0.95)**	F-TKIs+Lin	
0.20(0.08–0.52)	1.10(0.49–2.43)	1.09(0.47–2.52)	0.67(0.41–1.11)	0.30(0.20–0.43)	1.06(0.29–3.82)	CT

Abbreviations: For survival outcomes (OS, PFS), an HR below 1 favors the column-defining treatment. For safety (SAEs), an OR below 1 favors the column-defining treatment. Comparisons with differences of statistical significance (p<0.05) are highlighted in bold format. TKIs, tyrosine kinase inhibitor; F, first-generation; S, second-generation; Bev, bevacizumab; CT, chemotherapy; Lin, Linsitinib.

### Inconsistency assessment and treatment ranking

There were two independent closed loops in the network for PFS or OS: F-TKIs/S-TKIs/CT and F-TKIs/F-TKIs+CT/CT; one independent closed loop for SAEs: F-TKIs/S-TKIs/CT. Analysis of inconsistency showed that the NMA results were similar to the PWMA results for the three outcomes, which suggested the consistency between the direct and indirect evidence (S3 Fig).

**Fig 3:**
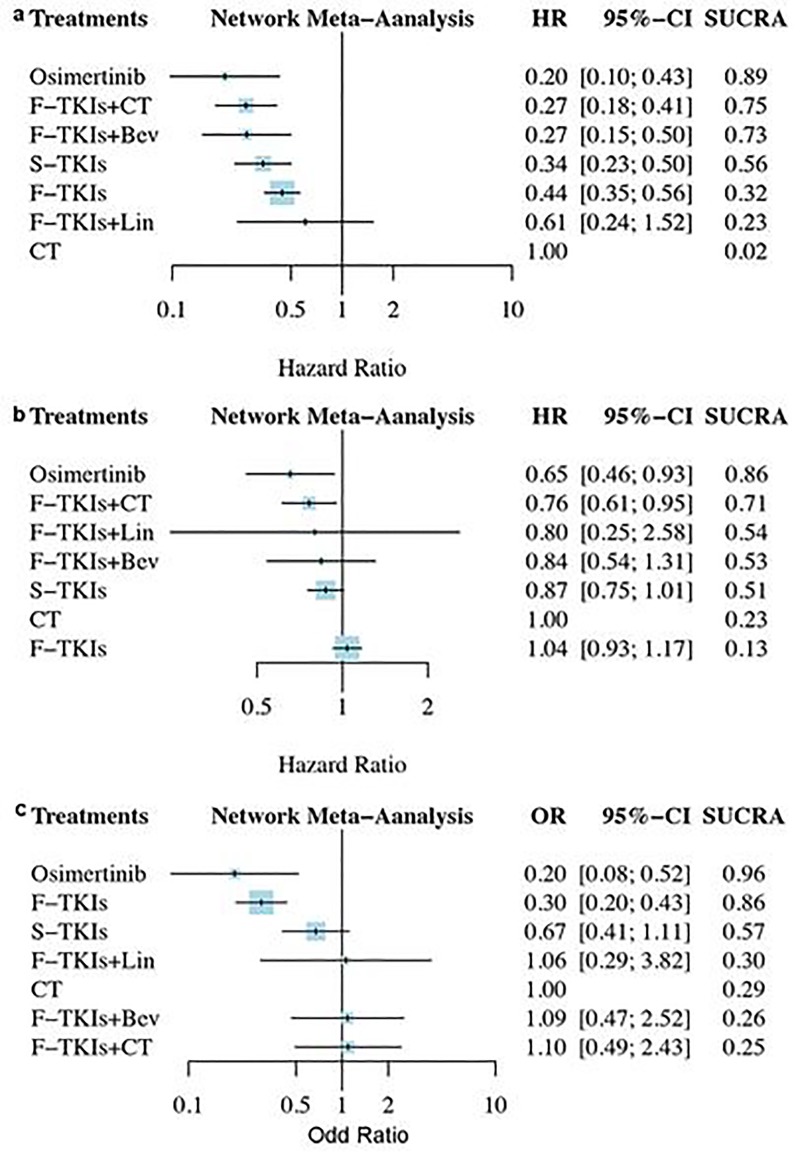
The treatment rankings based on SUCRA. (a) progression-free survival; (b) overall survival; (c) serious adverse events. SUCRA, surface under the cumulative ranking curves; TKIs, tyrosine kinase inhibitor; F, first-generation; S, second-generation; Bev, bevacizumab; CT, chemotherapy; Lin, Linsitinib.

The treatment rankings based on SUCRA are shown in Fig 3. In term of PFS (Fig 3A), osimertinib was the most effective treatment (0.89), followed by F-TKIs+CT (0.75), F-TKIs+Bev (0.73), and S-TKIs (0.56). With regard to OS (Fig 3B), osimertinib was still the most effective treatment (0.86), followed by F-TKIs+CT (0.71), F-TKIs+Lin (0.54), and F-TKIs+Bev (0.53). As for SAEs (Fig 3C), osimertinib was ranked as the least toxic regimen (0.96), followed by F-TKIs (0.86) and S-TKIs (0.57); F-TKIs+CT (0.25) was ranked as the highest toxic regimen.

**Fig 4:**
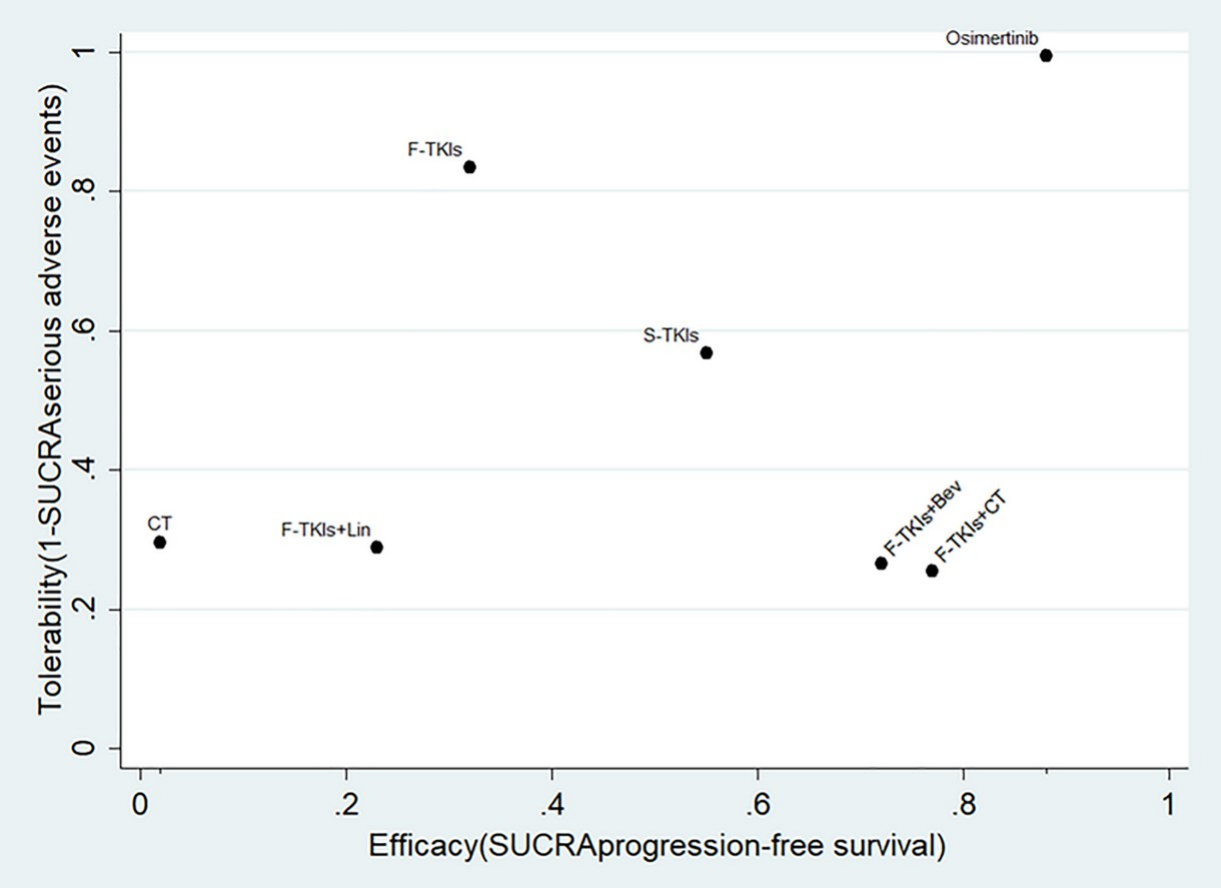
Ranking plot based simultaneously on efficacy (x-axis: SUCRA value of overall survival) and tolerability (y-axis: 1-SUCRA value of serious adverse events). SUCRA, surface under the cumulative ranking curves; TKIs, tyrosine kinase inhibitor; F, first-generation; S, second-generation; Bev, bevacizumab; CT, chemotherapy; Lin, Linsitinib.

To further assess the benefit-risk ratios of the seven treatments simultaneously, we ranked them based on the SUCRA values of PFS and tolerability (31-SUCRA _SAE_) in the ranking plot (Fig 4). Osimertinib was likely to be the optimal treatment because it had the most efficacy and best tolerability. F-TKIs+CT and F-TKIs+Bev were also two more effective regimens, but with relatively high risk of causing SAEs. S-TKIs achieved relatively good efficacy with moderate tolerability.

## Discussion

This novel network meta-analysis assessed the comparative efficacy and tolerability of all major TKIs based first-line treatments (including first-, second-, third-generation TKIs, and combination regimens involving TKIs) in advanced EGFR-mutated NSCLC. It showed that osimertinib provided significant PFS and OS advantage over F-TKIs, and had the highest probability of being the most effective treatment in improving PFS and OS, and with the best tolerability. In the FLAURA study [15] comparing first-line osimertinib with gefitinib or erlotinib in patients with advanced EGFR-mutated NSCLC, the median PFS (mPFS) was significantly longer with osimertinib than with standard F-TKIs (18.9 versus 10.0 months, *P* < 0.001). Overall SAEs were less frequent with osimertinib than with F-TKIs (34% vs. 45%).

F-TKIs+CT and F-TKIs+Bev were another two more effective regimens in our NMA. Based on treatment ranking, they were ranked second- and third- most effective regimen respectively, but with relatively high risk of causing SAEs. The NEJ009 study, a phase III trial evaluated the efficacy of a combination of gefitinib and CT in advanced NSCLC patients with EGFR mutations [18]. The combination arm demonstrated significantly improved mPFS (20.9 versus 11.2 months, *P* < 0.001) and median OS (mOS) (52.2 versus 38.8 months, P = 0.013) compared with gefitinib alone arm. A phase II study [20–21] comparing combination of erlotinib and bevacizumab with erlotinib alone in this patient population reported results with significant benefit on mPFS (16.4 versus 9.8 months, *P* = 0.0005). Similar improved mPFS was also observed in a phase III trial (NEJ026) [22] (16.9 months in erlotinib+bevacizumab arm versus 13.3 months in erlotinib alone arm, *P* < 0.001). Although both the two combination regimens were associated with higher incidence of grade 3 toxicities, few patients required dose reduction or withdrawal.

The survival difference between F-TKIs and S-TKIs has been investigated in two trials. LUX-LUNG 7 trial [11–12] showed no clinically meaningful survival benefit with afatinib versus with gefitinib in patients with advanced EGFR-mutated NSCLC. However, in another phase III trial [13–14] comparing dacomitinib with gefitinib in patients with EGFR-mutated NSCLC without brain metastases, dacomitinib was associated with significant improvement in mPFS (14.7 versus 9.2 months, *P* < 0.001) and mOS (34.1 months versus 26.8 months, *P* = 0.044) compared with gefitinib, but with increased grade 3 toxicities. In our NMA, S-TKIs was ranked fourth-most effective regimen with moderate risk of causing SAEs.

Based on the findings of this NMA, osimertinib seemed to be the preferable first-line treatment for patients with advanced EGFR-mutated NSCLC. However, the OS data were immature in the FLAURA study [15]. The survival rate at 18 months was not significantly longer with osimertinib than with F-TKIs (83% versus 71%) in the interim analysis [15]. More recently, postprogression outcomes of the FLAURA study have been reported [48]. Median second PFS was not reached [95% CI, 23.7-not calculable (NC)] in the osimertinib arm and 20.0 months (95% CI, 18.2-NC) in the standard-of-care (SoC) EGFR-TKI (gefitinib or erlotinib) arm [HR = 0.58, 95% CI: 0.44–0.78; *P* = 0.0004]. This suggested that osimertinib preserved clinical benefit after first progression. Moreover, median time to discontinuation of any EGFR-TKI or death was 23.0 months (95% CI, 19.5-NC) in the osimertinib arm and 16.0 months (95% CI, 14.8–18.6) in the SoC EGFR-TKI arm. These exploratory postprogression outcomes showed consistent improvements of osimertinib compared to SoC EGFR-TKI, and provide further confidence in the interim OS data.

There are several limitations in this network meta-analysis. First, in common with other meta-analyses, data were collected and analyzed basis of results reported from trials, and not on individual patient data. Therefore, effects of potential prognostic factors could not be accounted for. Second, most of included trials reported an immature OS data, and follow-up times across trials were different and generally short. Moreover, only one RCT investigated efficacy of osimertinib vs F-TKIs. These limitations do not allow us to reach a definitive conclusion about the superiority of one treatment over another. Third, this network meta-analysis included most of RCTs with Asians (15/25). We combined treatment effects for Asians and other groups assuming that there is no racial difference in the treatment effects. However, there was still no evidence supporting that the effect of TKIs among Asians is comparable to that among other racial groups, and we could not investigate such a racial difference in the treatment effect using this network meta-analysis data. Fourth, TKIs efficacy may be associated with patient characteristics (such as gender, race, and smoking status), tumor pathology, EGFR mutation types, and developing brain metastasis or not. However, we could not perform the subgroup-analyses because of insufficient data in the individual trials. Finally, some HRs of PFS or OS were calculated from the Kaplan–Meier curve due to that they were not directly reported in the articles. This may result in bias.

## Conclusions

Osimertinib seemed to be the most preferable first-line treatment in advanced EGFR-mutated NSCLC. However, limitations of the study including a single RCT investigating osimertinib and immature OS data need to be considered.

## Supporting information

S1 Fig Assessment of risk of bias. A: Methodological quality graph: authors’ judgment about each methodological quality item presented as percentages across all included studies; B: Methodological quality summary: authors’ judgment about each methodological quality item for each included study, “+” low risk of bias; “?” unclear risk of bias; “-” high risk of bias.
(TIF)

S2 Fig Comparison-adjusted funnel plots of publication bias test for progression-free survival. TKIs, tyrosine kinase inhibitor; F, first-generation; S, second-generation; Bev, bevacizumab; CT, chemotherapy; Lin, Linsitinib.
(TIF)

S3 Fig Inconsistency evaluation by node-splitting analyses. (a) progression-free survival; (b) overall survival; (c) serious adverse events. TKIs, tyrosine kinase inhibitor; F, first-generation; S, second-generation; CT, chemotherapy.
(TIF)

S1 Table PRISMA checklist. (DOC)

S2 Table Search strategy. (DOC)

S3 Table Demographic characteristics of included trials. (DOC)

S4 Table Results of individual trials. (DOC)
