# Supplementary figures and images for: First-line treatments in EGFR-mutated advanced non-small cell lung cancer: A network meta-analysis
Source: PLoS One. 2019 Oct 3;14(10):e0223530. doi: 10.1371/journal.pone.0223530 (PMC6776360; doi:10.1371/journal.pone.0223530)

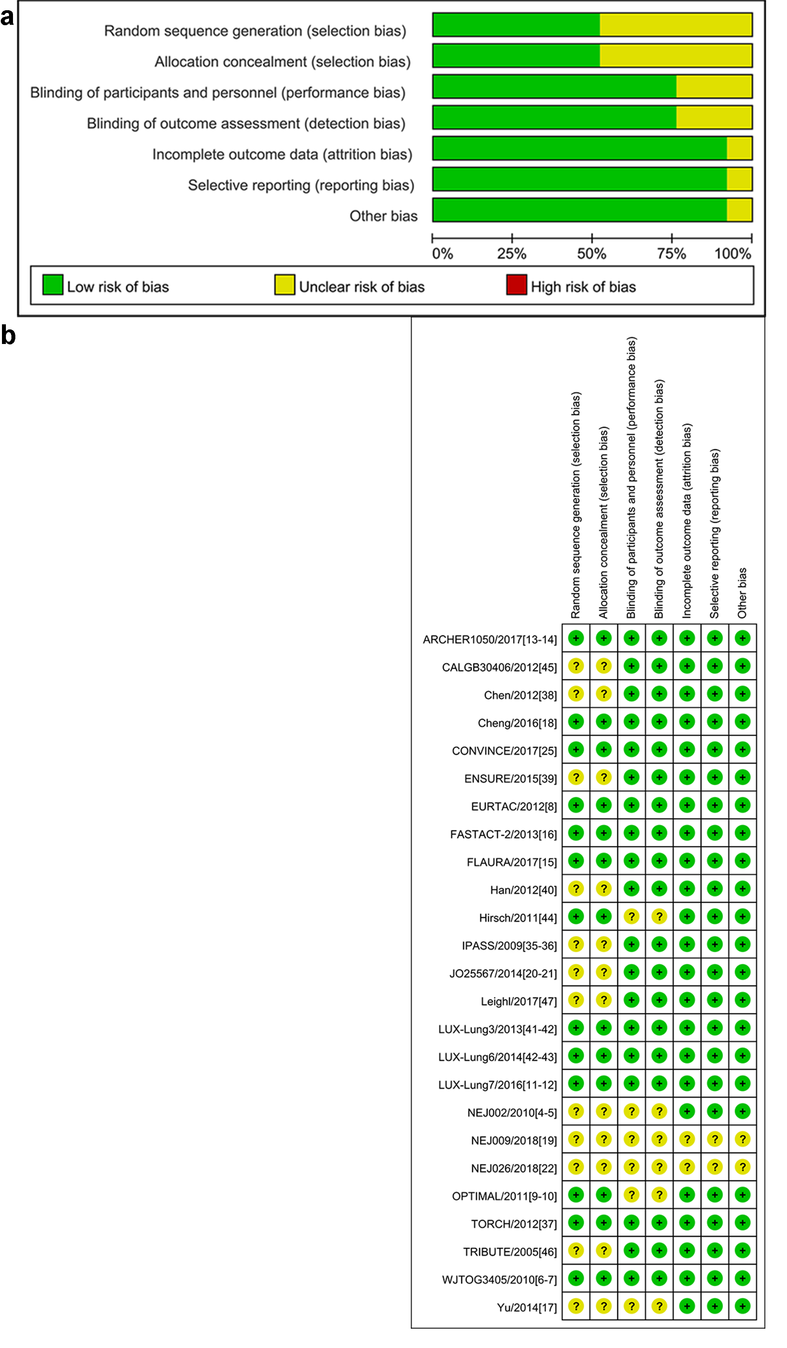

Supplement: S1 Fig — A: Methodological quality graph: authors’ judgment about each methodological quality item presented as percentages across all included studies; B: Methodological quality summary: authors’ judgment about each methodological quality item for each included study, “+” low risk of bias; “?” unclear risk of bias; “-” high risk of bias. (TIF) [file pone.0223530.s001.tif]

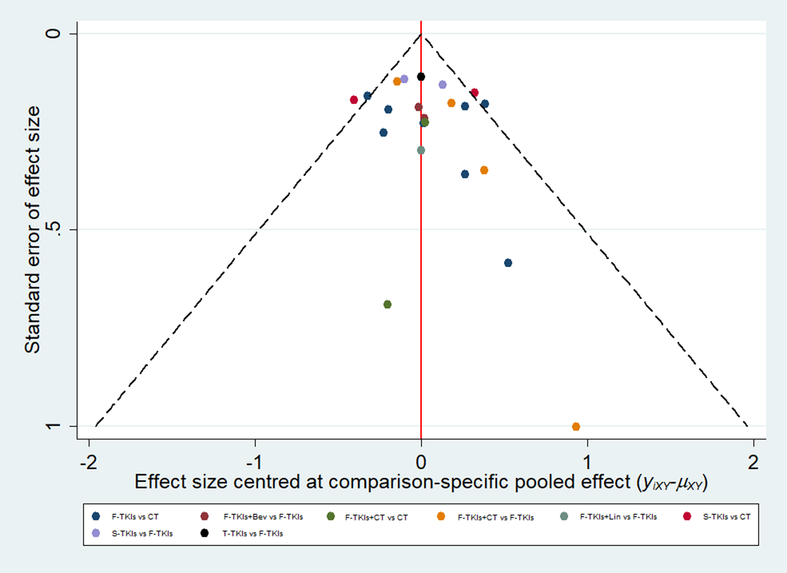

Supplement: S2 Fig — TKIs, tyrosine kinase inhibitor; F, first-generation; S, second-generation; Bev, bevacizumab; CT, chemotherapy; Lin, Linsitinib. (TIF) [file pone.0223530.s002.tif]

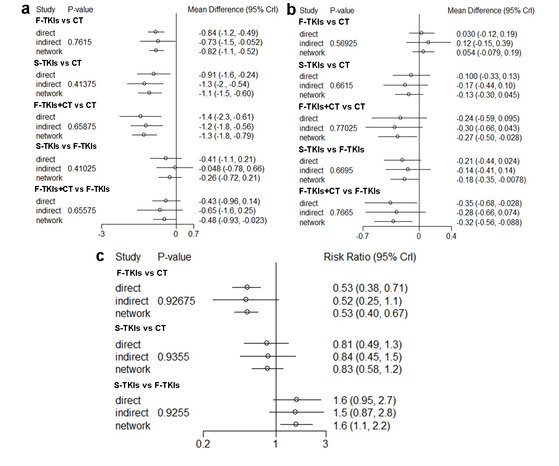

Supplement: S3 Fig — (a) progression-free survival; (b) overall survival; (c) serious adverse events. TKIs, tyrosine kinase inhibitor; F, first-generation; S, second-generation; CT, chemotherapy. (TIF) [file pone.0223530.s003.tif]
