# Supplementary material for: First-line treatments in EGFR-mutated advanced non-small cell lung cancer: A network meta-analysis
Source: PLoS One. 2019 Oct 3;14(10):e0223530. doi: 10.1371/journal.pone.0223530 (PMC6776360; doi:10.1371/journal.pone.0223530)
Supplement: S2 Table — (DOC) [file pone.0223530.s005.doc]

**S2 Table** Search strategy

**a:** Search strategy in PubMed

| # | Query |
| --- | --- |
| #1 | “Lung Neoplasms”[mh] |
| #2 | Lung Neoplasms[tiab] OR Neoplasms, Lung[tiab] OR Lung Neoplasm[tiab] OR Neoplasm, Lung[tiab] OR Neoplasms, Pulmonary[tiab] OR Neoplasm, Pulmonary[tiab] OR Pulmonary Neoplasm[tiab] OR Pulmonary Neoplasms[tiab] OR Lung Cancer[tiab] OR Cancer, Lung[tiab] OR Cancers, Lung[tiab] OR Lung Cancers[tiab] OR Pulmonary Cancer[tiab] OR Cancer, Pulmonary[tiab] OR Cancers, Pulmonary[tiab] OR Pulmonary Cancers[tiab] OR Cancer of the Lung[tiab] OR Cancer of Lung[tiab] |
| #3 | "Carcinoma, Non-Small-Cell Lung"[mh] |
| #4 | Carcinoma, Non Small Cell Lung[tiab] OR Carcinomas, Non-Small-Cell Lung[tiab] OR Lung Carcinoma, Non-Small-Cell[tiab] OR Lung Carcinomas, Non-Small-Cell[tiab] OR Non-Small-Cell Lung Carcinomas[tiab] OR Nonsmall Cell Lung Cancer[tiab] OR Non-Small-Cell Lung Carcinoma[tiab] OR Non Small Cell Lung Carcinoma[tiab] OR Carcinoma, Non-Small Cell Lung[tiab] OR Non-Small Cell Lung Cancer[tiab] OR NSCLC[tiab] |
| #5 | #1 OR #2 OR #3 OR #4 |
| #6 | Advanced[tiab] OR Stage IV[tiab] OR Stage 4[tiab] OR Stage four[tiab] OR StageIIIB[tiab] OR Metastatic[tiab] OR Metastases[tiab] |
| #7 | Kinase Inhibitor[tiab] OR Kinase Inhibitors[tiab] OR Gefitinib[tiab] OR Erlotinib[tiab] OR Icotinib[tiab] OR Afatinib[tiab] OR Dacomitinib[tiab] OR Osimertinib[tiab] |
| #8 | Chemotherapies[tiab] OR Chemotherapy[tiab] OR Docetaxel[tiab] OR Pemetrexed[tiab] OR Gemcitabine[tiab] OR Vinorelbine[tiab] OR Paclitaxel[tiab] |
| #9 | Bevacizumab[tiab] |
| #10 | #7 OR #8 OR #9 |
| #11 | First-line[tiab] OR Untreated[tiab] OR Chemotherapy naïve[tiab] OR Frontline[tiab] OR Treatment naïve[tiab] |
| #12 | Randomized Controlled Tial[pt] |
| #13 | Controlled Cinical Trial[pt] |
| #14 | Randomized[tiab] |
| #15 | Placebo[tiab] |
| #16 | Randomly[tiab] |
| #17  #18 | Trial[tiab]  Drug Therapy[sh] |
| #19 | Groups[tiab] |
| #20 | #12 OR #13 OR #14 OR #15 OR #16 OR #17 OR #18 OR #19 |
| #21 | Animals[mh] |
| #22 | Humans[mh] |
| #23 | #21 NOT #22 |
| #24 | #20 NOT #23 |
| #25 | #5 AND #6 AND #10 AND #11 AND #24 |

**b:** Search strategy in Embase

| # | Query |
| --- | --- |
| #1 | ‘lung cancer’/exp |
| #2 | ‘non small cell lung cancer’/exp |
| #3 | 'non small cell':ab,ti |
| #4 | ‘nsclc’:ti,ab |
| #5 | #1 OR #2 OR #3 OR #4 |
| #6 | ‘advanced':ab,ti OR ‘stage IV':ab,ti OR ‘stage 4':ab,ti OR ‘stage four':ab,ti OR ‘stageIIIB':ab,ti OR ‘metastatic':ab,ti OR ‘metastases':ab,ti |
| #7 | ‘kinase inhibitor':ab,ti OR ‘kinase Inhibitors':ab,ti OR ‘gefitinib':ab,ti OR ‘erlotinib':ab,ti OR ‘icotinib':ab,ti OR ‘afatinib':ab,ti OR ‘dacomitinib':ab,ti OR ‘osimertinib':ab,ti |
| #8 | 'chemoradi*':ab,ti OR ‘docetaxel':ab,ti OR ‘pemetrexed':ab,ti OR ‘gemcitabine':ab,ti OR ‘vinorelbine':ab,ti OR ‘paclitaxel':ab,ti |
| #9 | ‘bevacizumab':ab,ti |
| #10 | #7 OR #8 OR #9 |
| #11 | ‘first-line':ab,ti OR ‘untreated':ab,ti OR ‘chemotherapy naïve':ab,ti OR ‘frontline':ab,ti OR ‘treatment naïve':ab,ti |
| #12 | 'trial':ab,ti |
| #13 | 'random*':ab,ti |
| #14 | 'clinical trial'/de OR 'controlled clinical trial'/de OR 'randomized controlled trial'/de |
| #15 | #12 OR #13 OR #14 |
| #16 | #5 And #6 And #10 And #11 And #15 |

**c:** Search strategy in Cochrane Library

| # | Query |
| --- | --- |
| #1 | MeSH descriptor: [Carcinoma, Non-Small-Cell Lung] explode all trees |
| #2 | MeSH descriptor: [Lung Neoplasms] explode all trees |
| #3 | ((lung OR pulmon*) AND (neoplas* OR cancer OR carcinoma* OR tumour* or tumor*)) |
| #4 | non-small cell* |
| #5 | non small cell* |
| #6 | nonsmall cell* |
| #7 | Nsclc |
| #8 | #1 OR #2 OR #3 OR #4 OR #5 OR #6 OR #7 |
| #9 | (advanced OR stage IV OR stage 4 OR stage four OR stageIIIB OR metastatic OR metastases):ti,ab |
| #10 | (kinase inhibitor OR kinase Inhibitors OR gefitinib OR erlotinib OR icotinib OR afatinib OR dacomitinib OR osimertinib):ti,ab |
| #11 | (chemoradi* OR docetaxel OR pemetrexed OR gemcitabine OR vinorelbine OR paclitaxel):ti,ab |
| #12 | (bevacizumab):ti,ab |
| #13 | #10 OR #11 OR #12 |
| #14 | (first-line OR untreated OR chemotherapy naïve OR frontline OR treatment naïve):ti,ab |
| #15 | #8 AND #9 AND #13 AND #14 |

**d:** Search strategy in Web of Science

| # | Query |
| --- | --- |
| #1 | TS=("lung cancer" OR "non-small cell lung cancer" OR NSCLC OR ((lung OR pulmon*) AND (neoplas* OR cancer OR carcinoma* OR tumour* or tumor*))) |
| #2 | TS=("advanced” OR “stage IV” OR “stage 4” OR “stage four” OR “stageIIIB” OR “metastatic” OR “metastases”) |
| #3 | TS=(“kinase inhibitor” OR “kinase Inhibitors” OR “gefitinib” OR “erlotinib” OR “icotinib” OR “afatinib” OR “dacomitinib” OR “osimertinib”) |
| #4 | TS=("chemoradi*” OR “docetaxel” OR “pemetrexed” OR “gemcitabine” OR “vinorelbine” OR “paclitaxel”) |
| #5 | TS=(“bevacizumab”) |
| #6 | #3 OR #4 OR #5 |
| #7 | TS=(“first-line” OR “untreated” OR “chemotherapy naïve” OR “frontline” OR “treatment naïve”) |
| #8 | TS=("randomized controlled trial" OR "controlled clinical trial" OR "clinical trial" OR "random*" OR "rct*" OR "crossover" OR "masked” OR “blind*" OR "placebo*") |
| #9 | #1 AND #2 AND #6 AND #7 AND #8 |
