# Supplementary material for: First-line treatments in EGFR-mutated advanced non-small cell lung cancer: A network meta-analysis
Source: PLoS One. 2019 Oct 3;14(10):e0223530. doi: 10.1371/journal.pone.0223530 (PMC6776360; doi:10.1371/journal.pone.0223530)
Supplement: S3 Table — (DOC) [file pone.0223530.s006.doc]

**S3 Table Demographic characteristics of included trials.**

| Trial | Treatment | Median | Male | Smokers | Stage | Squamous | EGFR Mutation |
| --- | --- | --- | --- | --- | --- | --- | --- |
|  |  | Age | (%) | (%) | IV(%) | (%) | Type 19/21(%) |
| NEJ002/2010[4-5] | F-TKIs | 63.9 | 36.8 | 34.2 | 77.2 | 2.6 | 50.9/43.0 |
|  | CT | 62.6 | 36.0 | 42.1 | 73.7 | 1.8 | 51.8/42.1 |
| WJTOG3405/2010[6-7] | F-TKIs | 64 | 31.4 | 29.1 | 47.7 | 1.2 | 58.1/41.9 |
|  | CT | 64 | 30.2 | 33.7 | 47.7 | 0 | 43.0/57.0 |
| EURTAC/2012[8] | F-TKIs | 65 | 33.0 | 34.0 | 91.0 | 1.0 | 66.0/34.0 |
|  | CT | 65 | 22.0 | 28.0 | 94.0 | 0 | 67.0/33.0 |
| OPTIMAL/2011[9-10] | F-TKIs | 57 | 41.0 | 28.0 | 87.0 | 12.0 | 52.0/48.0 |
|  | CT | 59 | 40.0 | 31.0 | 93.0 | 14.0 | 54.0/46.0 |
| LUX-Lung7/2016[11-12] | S-TKIs | 63 | 43.0 | 34.0 | 95.0 | 1.0 | 58.0/42.0 |
|  | F-TKIs | 63 | 33.0 | 33.0 | 98.0 | 1.0 | 58.0/42.0 |
| ARCHER1050/2017[13-14] | S-TKIs | 62 | 36.0 | 36.0 | 81.0 | NR | 59.0/41.0 |
|  | F-TKIs | 61 | 44.0 | 36.0 | 81.0 | NR | 59.0/41.0 |
| FLAURA/2017[15] | Osimertinib | 64 | 36.0 | 35.0 | 95.0 | 1.0 | 63.0/37.0 |
|  | F-TKIs | 64 | 38.0 | 37.0 | 95.0 | 2.0 | 63.0/37.0 |
| FASTACT-2/2013[16] | F-TKIs+CT | NR | NR | NR | NR | NR | NR |
|  | CT | NR | NR | NR | NR | NR | NR |
| Yu/2014[17] | F-TKIs+CT | NR | NR | NR | NR | NR | NR |
|  | CT | NR | NR | NR | NR | NR | NR |
| Cheng/2016[18] | F-TKIs+CT | 62 | 35.0 | 36.0 | 83.0 | 0 | 52.0/41.0 |
|  | F-TKIs | 62 | 37.0 | 28.0 | 88.0 | 0 | 62.0/35.0 |
| NEJ009/2018[19] | F-TKIs+CT | 64.1 | 37.2 | 56.4 | 80.2 | 1.2 | NR |
|  | F-TKIs | 64.8 | 32.9 | 57.1 | 81.8 | 1.2 | NR |
| JO25567/2014[20-21] | F-TKIs+Bev | 67 | 40.0 | 44.0 | 80.0 | 1 | 53.0/47.0 |
|  | F-TKIs | 67 | 34.0 | 42.0 | 81.0 | 1 | 52.0/48.0 |
| NEJ026/2018[22] | F-TKIs+Bev | 67 | 36.6 | 42.0 | 73.2 | 1.8 | 50.0/50.0 |
|  | F-TKIs | 68 | 34.8 | 42.9 | 75.0 | 0 | 49.1/50.9 |
| CONVINCE/2017[25] | F-TKIs | 56 | 29.1 | 21.6 | 90.5 | 0 | 54.1/45.9 |
|  | CT | 56 | 30.7 | 21.2 | 89.1 | 0 | 54.0/46.0 |
| IPASS/2009[35-36] | F-TKIs | 57 | NR | NR | NR | NR | NR |
|  | CT | 57 | NR | NR | NR | NR | NR |
| TORCH/2012[37] | F-TKIs | NR | NR | NR | NR | NR | NR |
|  | CT | NR | NR | NR | NR | NR | NR |
| Chen/2012[38] | F-TKIs | NR | NR | NR | NR | NR | NR |
|  | CT | NR | NR | NR | NR | NR | NR |
| ENSURE/2015[39] | F-TKIs | 57.5 | 38.2 | 28.1 | 90.9 | 5.4 | 52.3/47.7 |
|  | CT | 56 | 39.3 | 30.9 | 93.5 | 5.5 | 57.0/43.0 |
| Han/2012[40] | F-TKIs | NR | NR | NR | NR | NR | NR |
|  | CT | NR | NR | NR | NR | NR | NR |
| LUX-Lung3/2013[41-42] | S-TKIs | 61.5 | 36.1 | 0 | 91.3 | 0 | 49.1/39.6 |
|  | CT | 61 | 33.0 | 0.9 | 85.2 | 0 | 49.6/40.9 |
| LUX-Lung6/2014[42-43] | S-TKIs | 58 | 36.0 | 25.2 | 93.4 | 0 | 51.2/38.0 |
|  | CT | 58 | 32.0 | 18.9 | 95.1 | 0 | 50.8/37.7 |
| Hirsch/2011[44] | F-TKIs+CT | NR | NR | NR | NR | NR | NR |
|  | F-TKIs | NR | NR | NR | NR | NR | NR |
| CALGB30406/2012[45] | F-TKIs+CT | NR | NR | NR | NR | NR | NR |
|  | F-TKIs | NR | NR | NR | NR | NR | NR |
| TRIBUTE/2005[46] | F-TKIs+CT | NR | NR | NR | NR | NR | NR |
|  | CT | NR | NR | NR | NR | NR | NR |
| Leighl/2017[47] | F-TKIs+Lin | 61.5 | 31.8 | 25.0 | 97.7 | 2.3 | 59.1/56.8 |
|  | F-TKIs | 57.5 | 27.3 | 27.3 | 100 | 0 | 40.9/43.2 |

Abbreviations: NR, not reported; TKIs, tyrosine kinase inhibitor; F, first-generation; S, second-generation; Bev, bevacizumab; CT, chemotherapy; Lin, Linsitinib.
