# Supplementary material for: First-line treatments in EGFR-mutated advanced non-small cell lung cancer: A network meta-analysis
Source: PLoS One. 2019 Oct 3;14(10):e0223530. doi: 10.1371/journal.pone.0223530 (PMC6776360; doi:10.1371/journal.pone.0223530)
Supplement: S4 Table — (DOC) [file pone.0223530.s007.doc]

**S4 Table Results of individual trials.**

| Trial | Treatment | PFS HR (95%CI) | OS HR (95%CI) | SAEs num (size) |
| --- | --- | --- | --- | --- |
| NEJ002/2010[4-5] | F-TKIs | 0.32(0.24-0.44) | 0.89(0.63-1.24) | 47(114) |
|  | CT | - | - | 81(113) |
| WJTOG3405/2010[6-7] | F-TKIs | 0.33(0.20-0.54) | NR | 46(87) |
|  | CT | - | - | 66(88) |
| EURTAC/2012[8] | F-TKIs | 0.34(0.23-0.49) | 1.04(0.65-1.68) | 38(84) |
|  | CT | - | - | 55(82) |
| OPTIMAL/2011[9-10] | F-TKIs | 0.16(0.10-0.26) | 1.19(0.83-1.71) | 14(83) |
|  | CT | - | - | 47(72) |
| LUX-Lung7/2016[11-12] | S-TKIs | 0.74(0.57-0.95) | 0.86(0.66-1.12) | 50(160) |
|  | F-TKIs | - | - | 29(159) |
| ARCHER1050/2017[13-14] | S-TKIs | 0.59(0.47-0.74) | 0.76(0.58-0.99) | 143(227) |
|  | F-TKIs | - | - | 92(224) |
| FLAURA/2017[15] | Osimertinib | 0.46(0.37-0.57) | 0.63(0.45-0.88) | 89(279) |
|  | F-TKIs | - | - | 114(277) |
| FASTACT-2/2013[16] | F-TKIs+CT | 0.25(0.16-0.39) | 0.48(0.27-0.84) | NR |
|  | CT | - | - | NR |
| Yu/2014[17] | F-TKIs+CT | 0.20(0.05-0.75) | NR | NR |
|  | CT | - | - | NR |
| Cheng/2016[18] | F-TKIs+CT | 0.68(0.48-0.96) | - | 53(126) |
|  | F-TKIs |  | - | 12(65) |
| NEJ009/2018[19] | F-TKIs+CT | 0.49(0.39-0.63) | 0.70(0.52-0.93) | 110(169) |
|  | F-TKIs | - | - | 54(172) |
| JO25567/2014[20-21] | F-TKIs+Bev | 0.63(0.41-0.96) | 0.81(0.53-1.23) | 68(75) |
|  | F-TKIs | - | - | 41(77) |
| NEJ026/2018[22] | F-TKIs+Bev | 0.61(0.42-0.88) | NR | 63(112) |
|  | F-TKIs | - | - | 43(114) |
| CONVINCE/2017[25] | F-TKIs | 0.61(0.43-0.87) | 0.97(0.72-1.31) | 14(148) |
|  | CT | - | - | 34(137) |
| IPASS/2009[35-36] | F-TKIs | 0.54(0.38-0.79) | 1.00(0.76-1.33) | NR |
|  | CT | - | - | NR |
| TORCH/2012[37] | F-TKIs | 1.71(0.82-3.60) | 1.58(0.7-3.57) | NR |
|  | CT | - | - | NR |
| Chen/2012[38] | F-TKIs | 0.70(0.22-2.18) | 2.17(0.45-10.37) | NR |
|  | CT | - | - | NR |
| ENSURE/2015[39] | F-TKIs | 0.42(0.27-0.66) | 0.91(0.63-1.31) | 39(110) |
|  | CT | - | - | 60(104) |
| Han/2012[40] | F-TKIs | 0.54(0.27-1.10) | 1.04(0.50-2.18) | NR |
|  | CT | - | - | NR |
| LUX-Lung3/2013[41-42] | S-TKIs | 0.58(0.43-0.78) | 0.88(0.66-1.17) | 140(229) |
|  | CT | - | - | 62(111) |
| LUX-Lung6/2014[42-43] | S-TKIs | 0.28(0.20-0.39) | 0.93(0.72-1.22) | 86(239) |
|  | CT | - | - | 68(113) |
| Hirsch/2011[44] | F-TKIs+CT | 1.44(0.20-10.15) | NR | NR |
|  | F-TKIs | - | - | NR |
| CALGB30406/2012[45] | F-TKIs+CT | 0.83(0.42-1.65) | 0.73(0.30-1.77) | NR |
|  | F-TKIs | - | - | NR |
| TRIBUTE/2005[46] | F-TKIs+CT | NR | 1.00(0.69-1.45) | NR |
|  | CT | - | - | NR |
| Leighl/2017[47] | F-TKIs+Lin | 1.37(0.76-2.45) | 0.77(0.24-2.42) | 22(43) |
|  | F-TKIs | - | - | 10(44) |

Abbreviations: PFS, progression-free survival; OS, overall survival; HR, hazard ratio; CI, confidence interval; SAEs, serious adverse events; Num, number; NR, not reported; TKIs, tyrosine kinase inhibitor; F, first-generation; S, second-generation; Bev, bevacizumab; CT, chemotherapy; Lin, Linsitinib. num, number of SAEs; size, sample size.
